# Edaravone Dexborneol in acute ischemic stroke patients treated with combined intravenous thrombolysis and mechanical thrombectomy for large vessel occlusion

**DOI:** 10.3389/fneur.2025.1681859

**Published:** 2025-10-21

**Authors:** Bo Peng, Ruilong Wang, Wei Jiang, Mo Zou, Hao Li, Hongxu Zhang

**Affiliations:** ^1^Department of Neurology, Dandong Central Hospital, Dandong, China; ^2^Department of Neurology, Harbin Jiarun Hospital, Harbin, China; ^3^Department of Respiratory, Harbin Jiarun Hospital, Harbin, China; ^4^Department of Neurology, Fuxin Tong’an Hospital, Fuxin, China; ^5^Department of Breast Surgery, Harbin Medical University Cancer Hospital, Harbin, China

**Keywords:** acute ischemic stroke, mechanical thrombectomy, intravenous thrombolysis, Edaravone Dexborneol, inflammatory biomarkers, clinical outcomes

## Abstract

**Background:**

Mechanical thrombectomy (MT), when combined with intravenous thrombolysis (IVT), has emerged as an effective therapeutic strategy for acute ischemic stroke (AIS). Edaravone Dexborneol, a novel fixed-dose combination of edaravone (30 mg) and dexborneol (7.5 mg) with dual free radical scavenger, has also demonstrated neuroprotective benefits in AIS management. This study aims to investigate the potential effects of Edaravone Dexborneol in patients with AIS who have undergone MT and IVT.

**Methods:**

This single-center retrospective cohort study enrolled 207 patients with AIS who received both IVT and MT between January 2019 and June 2024. Based on whether they received Edaravone Dexborneol treatment, patients were categorized into the Edaravone Dexborneol group and the control group. Baseline characteristics, inflammatory biomarkers, functional outcomes, mortality, and safety endpoints were compared.

**Results:**

Compared to the control group, the Edaravone Dexborneol group showed significantly lower levels of pro-inflammatory cytokines (IL-1β, IL-6, TNF-α) and higher levels of anti-inflammatory cytokines (IL-10, IL-35, TGF-β) at Days 3, 7, and 14 (all *p* < 0.01). Functionally, a greater proportion of patients in the Edaravone Dexborneol group achieved favorable outcomes (mRS 0–2) both at discharge (51.82% vs. 35.05%, *p* = 0.017) and at 90 days (67.27% vs. 46.39%, *p* = 0.003). All-cause mortality was numerically lower both in-hospital and at 90 days, without an increase in major safety events, including symptomatic intracranial hemorrhage.

**Conclusion:**

Patients treated with Edaravone Dexborneol were associated with better functional outcomes three months after stroke onset compared to those who did not receive the treatment. These findings may be related to the drug’s anti-inflammatory properties; however, randomized controlled trials are needed to confirm efficacy in patients undergoing MT combined with IVT.

## Introduction

According to current guidelines, patients with anterior circulation stroke caused by large vessel occlusion (LVO) should receive intravenous thrombolysis (IVT) followed by mechanical thrombectomy (MT) within a 4.5-h window (1, 2). Although reperfusion therapies for ischemic stroke have advanced considerably, approximately 50% of acute ischemic stroke (AIS) patients still experience clinically ineffective reperfusion (3).

Emerging evidence suggests that inflammation plays a critical role in clinically ineffective reperfusion following successful recanalization in AIS. Although large-vessel patency is restored, persistent microvascular obstruction—often termed the “no-reflow phenomenon”—can result from leukocyte plugging, platelet microthrombi, and endothelial swelling, all of which are exacerbated by inflammatory responses (4). Moreover, reperfusion itself triggers oxidative stress and activates immune cascades, leading to secondary brain injury and disruption of the blood–brain barrier. Elevated levels of proinflammatory cytokines such as IL-6, TNF-α, and IL-1β have been associated with poor neurological outcomes, even in patients with technically successful thrombectomy (5, 6). These findings indicate that neuroinflammation may significantly compromise the clinical benefits of reperfusion therapies, and targeting post-recanalization inflammation is increasingly recognized as a promising therapeutic strategy.

Emerging evidence from clinical cohorts of AIS has demonstrated Edaravone Dexborneol has been reported to possess both anti-inflammatory and potential neuroprotective properties (7, 8). However, despite these advances, there remains a critical gap in knowledge. Specifically, the clinical efficacy of Edaravone Dexborneol in patients receiving both intravenous thrombolysis and mechanical thrombectomy has not been clearly established. Given the high prevalence of ineffective reperfusion and inflammation-mediated injury after recanalization, determining whether Edaravone Dexborneol confers additional benefit in this high-risk population is of considerable clinical importance.

Therefore, the present study aimed to evaluate the therapeutic impact of Edaravone Dexborneol on functional outcomes and inflammatory responses in AIS patients undergoing combined IVT and MT. We hypothesized that Edaravone Dexborneol would improve functional recovery at 90 days by attenuating neuroinflammation and reducing reperfusion-related injury.

## Methods

### Study population

Between January 2019 and June 2024, all consecutive AIS patients treated with MT with IVT in a single tertiary institute were retrospectively collected using a standardized case report form including clinical information, radiological findings, and outcomes.

Inclusion criteria were adapted from previous literature (1) and included the following:

1. Acute ischemic stroke caused by anterior circulation large vessel occlusion (LVO), specifically involving the internal carotid artery (ICA) or proximal middle cerebral artery (M1) segment, confirmed by CT angiography (CTA) or MR angiography (MRA);
2. Age ≥ 18 years;
3. Intravenous administration of alteplase (0.9 mg/kg body weight) within 4.5 h from symptom onset;
4. MT performed within 6 h from symptom onset;
5. Baseline National Institutes of Health Stroke Scale (NIHSS) score ≥ 6;
6. Alberta Stroke Program Early CT Score (ASPECTS) ≥ 6 on initial non-contrast CT scan.

Patients were excluded if they met any of the following criteria:

1. Posterior circulation stroke, or occlusions located in non-target vessels (e.g., basilar artery, posterior cerebral artery, or M2/M3 branches not extending to the M1 segment).
2. Age < 18 years, or legal inability to provide consent for data usage (if applicable in the retrospective study design).
3. Pre-stroke functional disability, defined as a modified Rankin Scale (mRS) score > 2, indicating significant baseline impairment.
4. NIH Stroke Scale (NIHSS) score < 6, suggesting mild neurological deficits and uncertain benefit from thrombectomy.
5. ASPECTS < 6 on initial non-contrast CT scan, indicating extensive early ischemic changes and limited salvageable brain tissue.
6. Time from symptom onset > 6 h at the time of arterial puncture, unless patients fulfilled extended-window criteria (e.g., DAWN or DEFUSE-3) and were included as a predefined subgroup.
7. Contraindications to intravenous thrombolysis, including:
  1. Active internal bleeding or known bleeding diathesis
  2. Platelet count < 100,000/mm^3^
  3. International standardized ratio (INR) > 1.7 or recent use of direct oral anticoagulants
  4. History of intracranial hemorrhage or recent major surgery (within 14 days)
  5. Severe uncontrolled hypertension (>185/110 mmHg at the time of treatment).
8. Failure to receive both IV alteplase and MT, i.e., patients who received only one modality or neither.
9. Incomplete clinical, imaging, or follow-up data, rendering outcome assessment infeasible.
10. Poor-quality baseline imaging, such as non-diagnostic computer tomography (CT) or magnetic resonance imaging (MRI), or inability to confirm the occlusion site.

**Figure 1:**
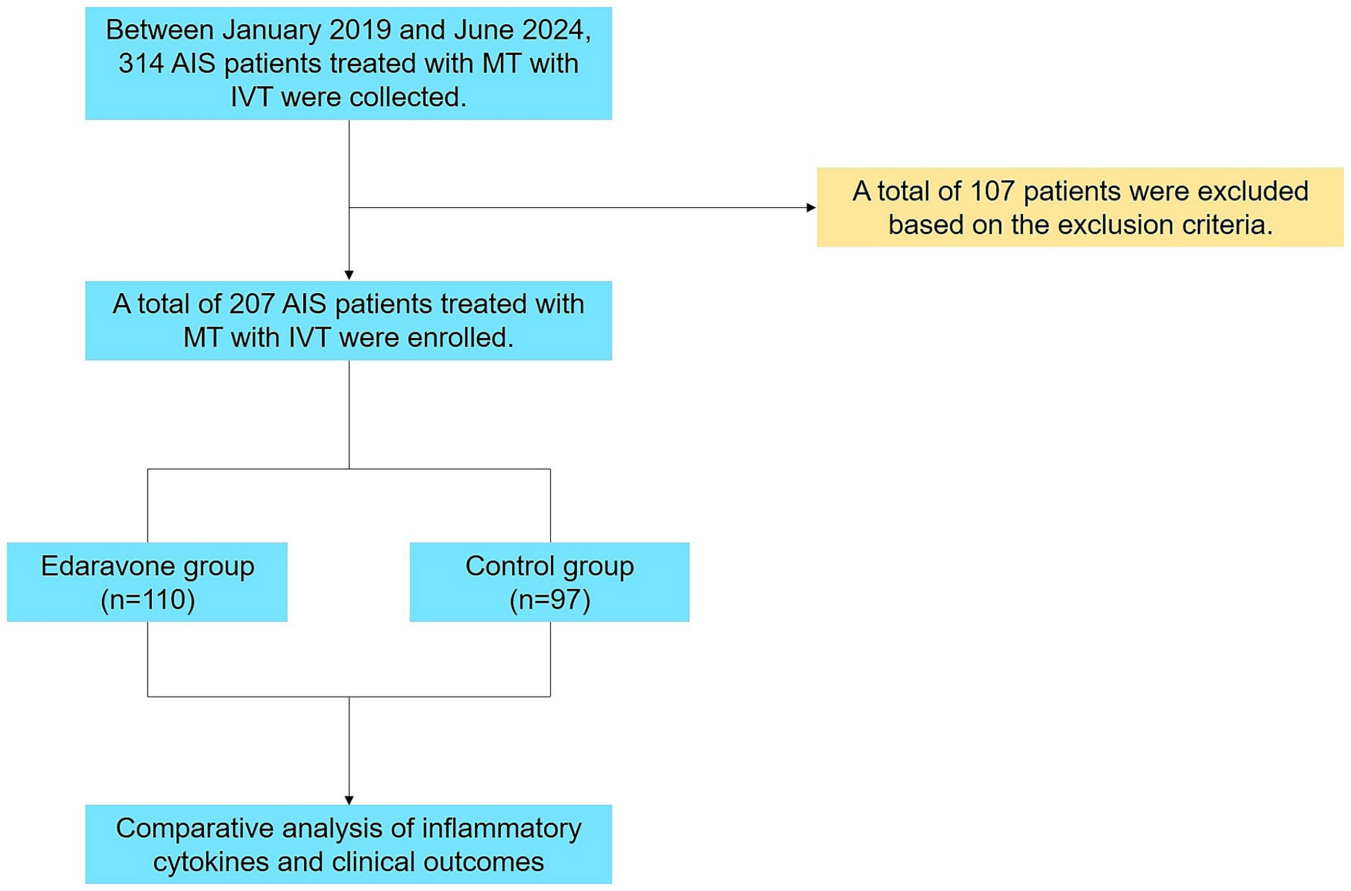
Study flowchart. AIS, acute ischemic stroke; MT, mechanical thrombectomy; IVT, intravenous thrombolysis.

After applying the inclusion and exclusion criteria, a total of 207 patients with AIS were ultimately enrolled in this study (Figure 1). All the eligible patients were divided into two groups according to whether Edaravone Dexborneol was administered during hospitalization. The decision to prescribe Edaravone Dexborneol was made by the treating physician based on individual clinical judgment and treatment practices at the time, rather than patient consent or random allocation.

### Procedures

All patients underwent standardized treatment according to AIS guidelines (1). The standardized group included antiplatelet aggregation or anticoagulant therapy, statin therapy, and control of risk factors regarding AIS. The treatment group received intravenous infusion of Edaravone Dexborneol (37.5 mg per dose, consisting of edaravone 30 mg + dexborneol 7.5 mg, Simcere Pharmaceutical, China) administered by neurological nurses every 12 h for 14 days or hospital discharge and the standardized treatment. The first dose was administered immediately after IVT infusion and before groin puncture for MT; in patients without IVT, the first dose was given immediately after vascular access and prior to reperfusion. Treatment adherence was high, with a median duration of 13 days (IQR 11–14). Early discontinuation occurred in 6 patients (5.5%), due to early discharge or mild adverse events.

### Clinical and radiologic evaluations

All patients underwent non-contrast CT or MRI; as part of the preprocedural evaluation. Baseline clinical data—including demographics, vascular risk factors, time of symptom onset, and initial neurological presentation—were collected in the emergency department. This included assessment of stroke severity using NIHSS and early ischemic changes using ASPECTS Score.

For patients with relative contraindications to IVT—such as advanced age or recent myocardial infarction (9)—the decision to proceed with IVT prior to MT was made at the discretion of the treating neurologist. This decision was based on radiologic findings, clinical presentation, and time from symptom onset. MT procedures were performed collaboratively by one interventional neuroradiologist and two interventional neurosurgeons, following consensus between the treating neurologist and the interventional team.

Successful reperfusion was defined as achieving a Thrombolysis in Cerebral Infarction (TICI) score of 2b or 3 on final angiographic imaging. Postprocedural intracranial hemorrhage (ICH) was assessed by follow-up CT within 24 h. Any ICH was defined as any hemorrhagic finding on follow-up CT within 24 h after reperfusion therapy (10). Symptomatic ICH was defined as any hemorrhage associated with a ≥ 4-point increase in the NIHSS score or resulting in death (11, 12). Hemorrhagic infarction (HI) was defined as petechial bleeding within the infarcted area without mass effect, classified per ECASS (13). Parenchymal hematoma (PH-1/PH-2): Homogeneous hematoma involving infarcted tissue. PH-1 was defined as <30% of the infarcted area with mild space-occupying effect; PH-2 was defined as ≥30% of the infarcted area with substantial mass effect (13). Early neurological deterioration (END): A ≥ 4-point increase in NIHSS score within 72 h of treatment (14). Renal dysfunction: New-onset acute kidney injury during hospitalization, defined as either a ≥ 50% increase in serum creatinine from baseline or an absolute increase ≥0.3 mg/dL (26.5 μmol/L) within 48 h (KDIGO criteria) (15). Efficacy outcomes were evaluated at 90 days using modified Rankin Scale (mRS), with a good functional outcome defined as a mRS score of ≤ 2 (16).

### Biomarkers of inflammatory response

This was a retrospective study utilizing prospectively collected clinical data and biological specimens at our medical center. Inflammatory biomarkers (IL-1β, IL-6, IL-10, IL-35, TNF-α, and TGF-β) are routinely measured in patients admitted with acute ischemic stroke, as part of standardized laboratory protocols established for both clinical monitoring and research purposes. Whole blood samples were collected at baseline (upon admission) and subsequently on days 3, 7, and 14. Plasma was separated by centrifugation and stored at −80 °C until analysis. The concentrations of inflammatory cytokines, including IL-1β, IL-6, IL-10, IL-35, TNF-α, and TGF-β, were quantified using a Cytokine Detection Kit (Raisecare, Qingdao, China) and analyzed on a Navios flow cytometer (Beckman Coulter, California, USA), following the manufacturer’s instructions. Laboratory staff conducting biomarker assays were blinded to patient group allocation to reduce potential measurement bias.

### Outcomes

The primary outcome was functional independence, defined as a modified Rankin Scale (mRS) score of 0–2 at 90 days follow-up. Secondary outcomes included functional status (mRS score), all-cause mortality, and safety outcomes such as symptomatic intracranial hemorrhage. Exploratory outcomes comprised longitudinal changes in inflammatory cytokines (IL-1β, IL-6, IL-10, IL-35, TNF-α, TGF-β) measured at baseline, day 3, day 7, and day 14, with the aim of providing mechanistic insights into the potential anti-inflammatory effects of Edaravone Dexborneol.

### Statistical analysis

Quantitative data were expressed as mean value ± standard deviation or median (interquartile range) if not normally distributed, while qualitative data were expressed as frequency (percentage). The independent two-sample *t*-test was used for between-group comparisons. Categorical variables were compared using the chi-square test or Fisher’s exact test, as appropriate. Mann–Whitney *U* tests were used to compare sets of non-normally distributed data. For mRS 0–2 at 90 days, logistic regression models were used, including both unadjusted and multivariable adjusted analyses. To further assess the ordinal nature of the mRS, proportional-odds ordinal logistic regression was performed for the full distribution of mRS scores (0–6). Cytokine levels were analyzed using linear mixed-effects models with a random intercept for each subject and fixed effects for group, time, and group × time interaction. Within-group changes from baseline were further evaluated using paired tests. *p*-values for the primary outcome were interpreted as confirmatory. Analyses of secondary and exploratory outcomes were considered descriptive; *p*-values were reported for completeness but were not adjusted for multiplicity, and findings were interpreted with caution to avoid overstatement of significance. A two-sided p-value < 0.05 was considered statistically significant for the primary outcome. The primary analysis followed an intention-to-treat (ITT) approach. A per-protocol (PP) sensitivity analysis excluding patients who discontinued treatment before Day 7 was also conducted, and results were consistent with the ITT analysis. All statistical analyses were conducted using SPSS software, version 22.0 (SPSS Inc., Chicago, IL, USA).

## Results

### Baseline characteristics

A total of 207 patients were included in the final analysis, with 110 patients receiving Edaravone Dexborneol in addition to standardized treatment (Edaravone Dexborneol group) and 97 patients receiving only the standardized treatment (Control group) (Figure 1). Baseline characteristics showed no significant differences between the two groups (Tables 1, 2). The median treatment duration in the Edaravone Dexborneol group was 13 days (IQR 11–14). Six patients (5.5%) discontinued early, but inclusion of these cases in the ITT analysis did not alter the overall findings.

**Table 1:** Patient characteristics.

Characteristics	Edaravone Dexborneol group (*n* = 110)	Control group (*n* = 97)	*p*-value
Age, years	70.61 ± 13.13	69.78 ± 13.42	0.174
Gender, *n* (%)			
Male	70 (63.64)	58 (59.79)	0.671
Female	40 (36.36)	39 (40.21)	0.671
Weight, kg	75.41 ± 7.67	75.24 ± 7.33	0.787
BMI, kg/m^2^	26.14 ± 3.12	25.66 ± 3.14	0.126
Medical history, *n* (%)			
Previous ischemic stroke	17 (15.45)	14 (14.43)	0.848
Hypertension	73 (66.36)	63 (64.95)	0.884
Hyperlipidemia	91 (82.73)	82 (84.54)	0.851
Diabetes	23 (20.91)	21 (21.65)	1.000
Coronary artery disease	15 (13.64)	14 (14.43)	1.000
Atrial fibrillation	21 (19.09)	21 (21.65)	0.730
Current smoker, *n* (%)	51 (46.36)	40 (41.24)	0.485
ASPECTS score	8 (7–9)	8 (7–9)	0.665
NIHSS score	12 (8–17)	11 (7–16)	0.334
Medications, *n* (%)			
Antihypertensive drugs	53 (48.2)	47 (48.5)	1.000
Statin or other lipid-lowering drug	87 (79.1)	76 (78.4)	1.000
Aspirin or other antiplatelet drug	89 (80.9)	80 (82.5)	0.858
Anticoagulation drug	18 (16.4)	20 (20.6)	0.475

Mean values ± standard deviation, median (interquartile range), and *n* (%) were reported for variables, respectively. BMI, body mass index; NIHSS, National Institutes of Health Stroke Scale; ASPECTS, Alberta Stroke Program Early CT Score.

**Table 2:** Characteristics of laboratory and catheterization room.

Characteristics	Edaravone Dexborneol group (*n* = 110)	Control group (*n* = 97)	*p*-value
Blood pressure, mmHg			
Systolic	150.61 ± 24.78	148.87 ± 19.21	0.545
Diastolic	88.71 ± 13.09	88.44 ± 11.83	0.866
Blood glucose, mmol/L	8.13 ± 2.45	7.88 ± 2.34	0.343
Median duration, min			
Onset-to-needle	59.92 ± 19.82	57.93 ± 20.80	0.435
Onset-to-puncture	260.3 ± 55.4	265.7 ± 60.5	0.275
Onset-to-recanalization	310.8 ± 60.3	315.7 ± 62.4	0.332
Cause of large-vessel occlusion, *n* (%)			
Intracranial atherosclerosis	46 (41.82)	38 (39.18)	0.885
Extracranial atherosclerosis	31 (28.18)	29 (29.90)	0.878
Cardioembolism	33 (30.0)	30 (30.93)	1.000
Anesthesia type, *n* (%)			
General anesthesia	30 (27.27)	31 (31.96)	0.541
Conscious sedation	80 (72.73)	66 (68.04)	0.541
Number of device passes, times	1.95 ± 0.90	1.92 ± 0.96	0.787
mTICI score at the end of the procedure, *n* (%)			
2b	46 (41.82)	37 (38.14)	0.313
2c	31 (28.18)	29 (29.90)	0.878
3	33 (30.0)	31 (31.96)	0.766

Mean values ± standard deviation, median (interquartile range), and *n* (%) were reported for variables, respectively. mTICI, modified thrombolysis in cerebral infarction.

### Levels and changes of inflammatory markers

**Figure 2:**
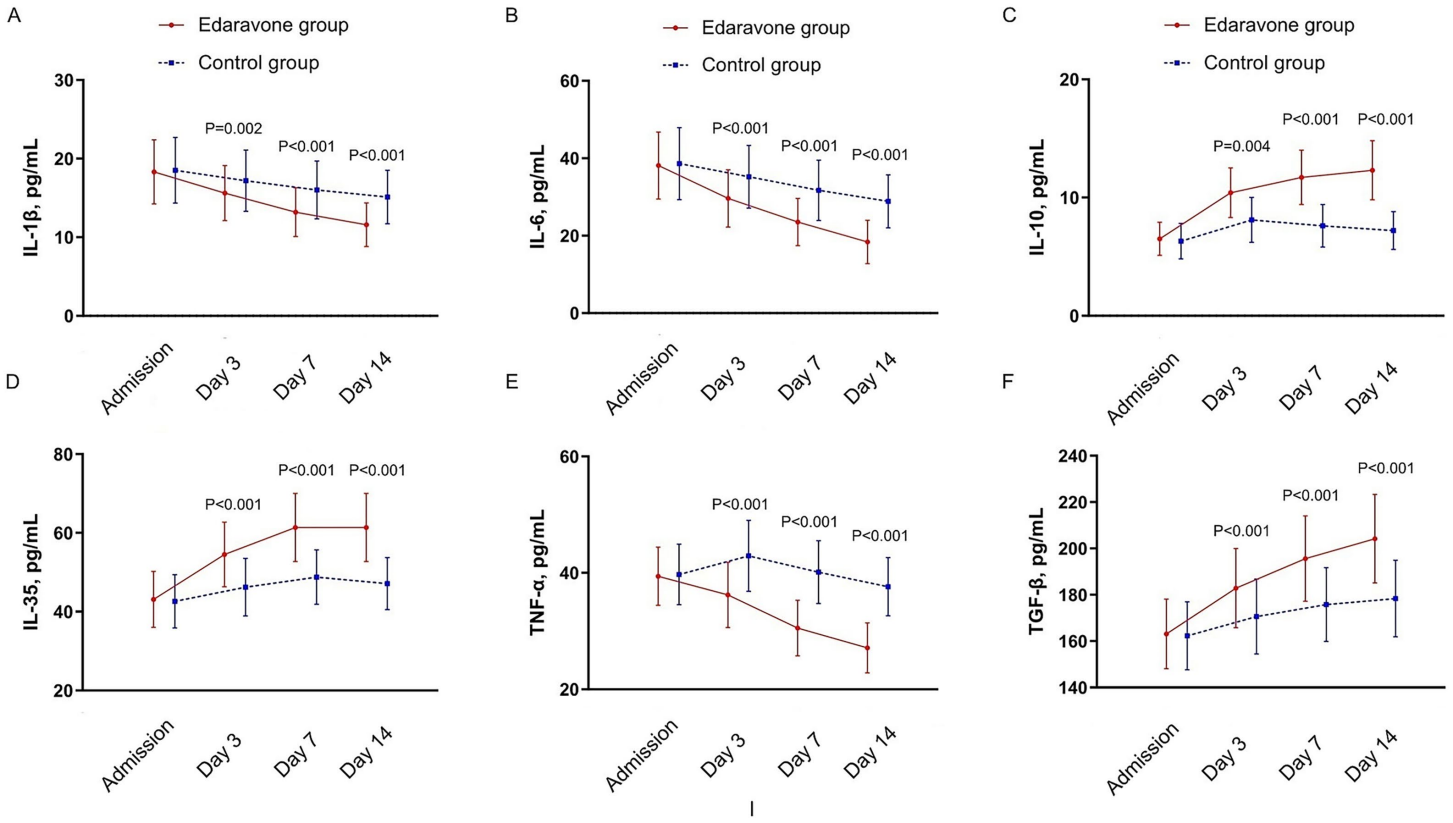
Levels and changes of inflammatory markers over time. **(A)** IL-1β; **(B)** IL-6; **(C)** IL-10; **(D)** IL-35; **(E)** TNF-α; **(F)** TGF-β; IL, interleukin; TNF, tumor necrosis factor; TGF, transforming growth factor. Blood samples were collected at multiple time points: at admission, Day 3, Day 7, and Day 14.

Table 3 and Figure 2 present the levels and temporal changes of various inflammatory markers, all of which exhibited variable trends over time. In the LMM analysis, significant main effects of group, time, and group × time interaction were observed for IL-1β, IL-6, IL-10, IL-35, TNF-α, and TGF-β (all *p* < 0.05), indicating both overall differences between groups and divergent temporal trends. Specifically, compared with baseline, patients receiving Edaravone Dexborneol exhibited greater reductions in pro-inflammatory cytokines (IL-1β, IL-6, TNF-α) and greater increases in anti-inflammatory cytokines (IL-10, IL-35, TGF-β) at days 3, 7, and 14 (all within-group *p* < 0.05). In contrast, the Control group showed only modest or no significant changes from baseline over the same period. These findings suggest that Edaravone Dexborneol treatment was associated with a more favorable modulation of inflammatory responses over time. Detailed mean values, standard deviations, and *p*-values for group, time, and group×time effects are reported in Table 3. Missing cytokine values (6–9% across timepoints) were due to unavailable or hemolyzed blood samples and were balanced between groups. Analyses using multiple imputation produced results consistent with the primary LMM models (data not shown), confirming the robustness of the findings.

**Table 3:** Longitudinal changes in cytokine levels with linear mixed-effects model results.

Cytokine (pg/mL)	Timepoint	Edaravone Dexborneol group (*n* = 110)	Control group (*n* = 97)	Group main effect (*P*)	Time main effect (*P*)	Group × time interaction (*P*)	Δ from baseline (within-group *P*)^*^
IL-1β	Baseline	18.31 ± 4.14	18.45 ± 4.21	0.031	<0.001	0.002	Day 3: <0.001; Day 7: <0.001; Day 14: <0.001
	Day 3	15.62 ± 3.45	17.21 ± 3.89				
	Day 7	13.21 ± 3.14	16.02 ± 3.67				
	Day 14	11.62 ± 2.78	15.12 ± 3.42				
IL-6	Baseline	38.11 ± 8.67	38.61 ± 9.32	0.027	<0.001	0.003	Day 3: <0.001; Day 7: <0.001; Day 14: <0.001
	Day 3	29.62 ± 7.41	35.21 ± 8.08				
	Day 7	23.45 ± 6.12	31.71 ± 7.78				
	Day 14	18.42 ± 5.56	28.89 ± 6.89				
IL-10	Baseline	6.51 ± 1.42	6.32 ± 1.45	0.012	<0.001	<0.001	Day 3: <0.001; Day 7: <0.001; Day 14: <0.001
	Day 3	10.41 ± 2.13	8.14 ± 1.89				
	Day 7	11.67 ± 2.31	7.56 ± 1.78				
	Day 14	12.31 ± 2.45	7.22 ± 1.56				
IL-35	Baseline	43.12 ± 7.12	42.62 ± 6.78	0.021	<0.001	0.001	Day 3: <0.001; Day 7: <0.001; Day 14: <0.001
	Day 3	54.45 ± 8.22	46.21 ± 7.31				
	Day 7	61.43 ± 8.71	48.78 ± 6.89				
	Day 14	61.42 ± 8.72	47.13 ± 6.56				
TNF-α	Baseline	39.41 ± 5.02	39.67 ± 5.21	0.019	<0.001	<0.001	Day 3: <0.001; Day 7: <0.001; Day 14: <0.001
	Day 3	36.21 ± 5.56	42.89 ± 6.12				
	Day 7	30.51 ± 4.82	40.13 ± 5.42				
	Day 14	27.13 ± 4.31	37.56 ± 5.02				
TGF-β	Baseline	163.12 ± 15.02	162.32 ± 14.71	0.035	<0.001	0.004	Day 3: <0.001; Day 7: <0.001; Day 14: <0.001
	Day 3	182.89 ± 17.12	170.62 ± 16.21				
	Day 7	195.56 ± 18.41	175.78 ± 15.89				
	Day 14	204.21 ± 19.12	178.41 ± 16.52				

^*^Δ from baseline: within-group change compared with baseline by paired t-test (or Wilcoxon signed-rank test if not normally distributed).

Values are mean ± SD.

Linear mixed-effects models included random intercepts for subjects and fixed effects for group, time, and group×time interaction.

Reported *p*-values are from fixed effects in the LMM.

Within-group change from baseline was additionally analyzed by paired tests at each follow-up time point.

IL, interleukin; TNF, tumor necrosis factor; TGF, transforming growth factor.

### Neurological function and primary outcomes

**Figure 3:**
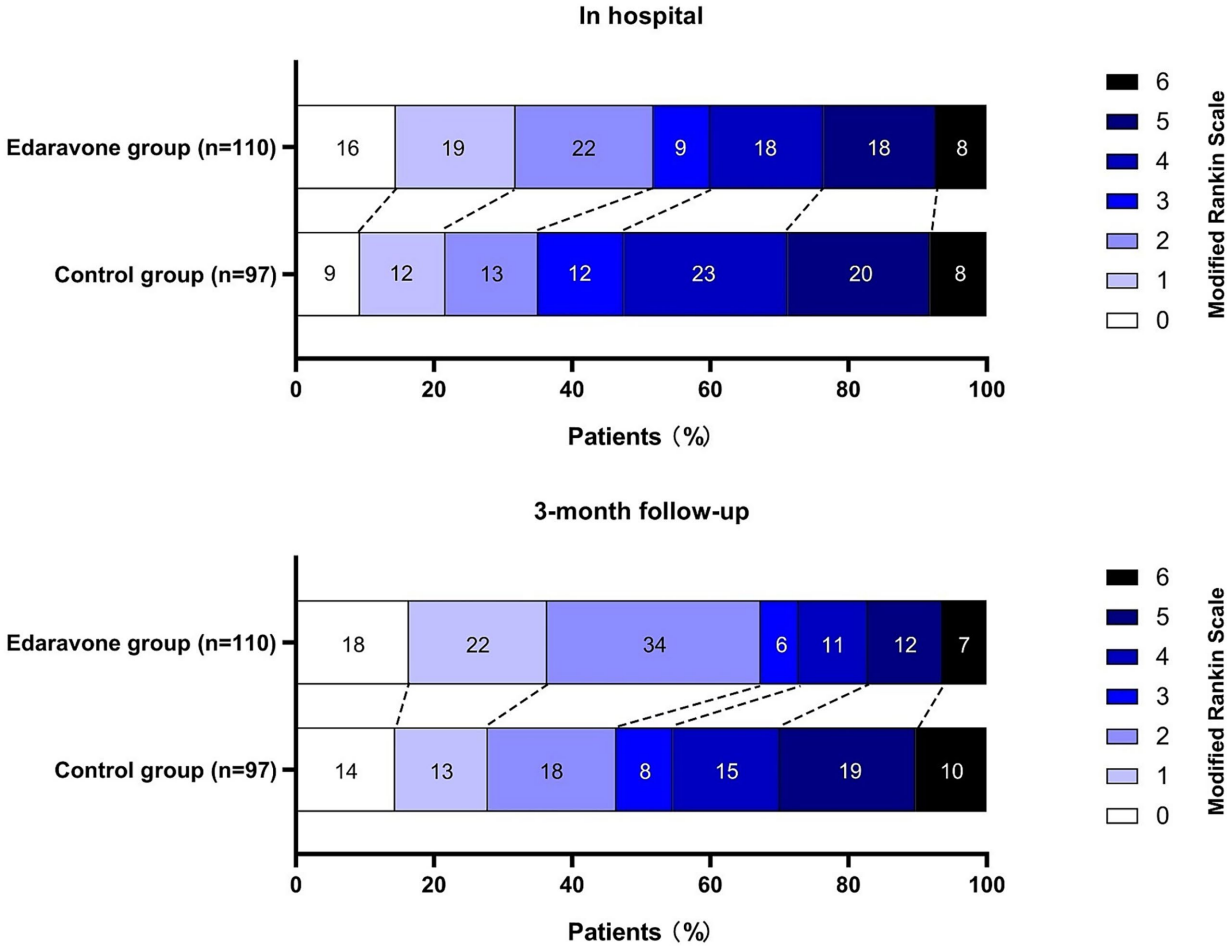
Scores on the modified Rankin Scale at discharge and follow-up.

At 90 days, the Edaravone Dexborneol group had a significantly lower median mRS score than the Control group [2 (IQR 1–4) vs. 3 (1–5); *p* = 0.018]. In unadjusted analyses, a higher proportion of patients achieved mRS 0–2 in the Edaravone Dexborneol group than in the Control group (67.3% vs. 46.4%; *p* = 0.003) (Table 4 and Figure 3). Multivariable logistic regression identified age [odds ratio (OR) 0.971; 95% confidence interval (CI), 0.943–0.999; *p* = 0.028], Edaravone Dexborneol (OR 2.134; 95% CI, 1.142–3.985; *p* = 0.018), internal carotid artery (ICA) occlusion (OR 0.381; 95% CI, 0.199–0.726; *p* = 0.004), and Onset-to-puncture (OR 0.988; 95% CI, 0.974–0.999; *p* = 0.011) as independent predictors of mRS 0–2 at 90 days follow-up. These findings confirm the primary hypothesis that Edaravone Dexborneol use is associated with better functional recovery at 90 days (Table 5). We additionally performed a proportional-odds ordinal logistic regression to evaluate the full distribution of 90-day mRS (0–6). The model was adjusted for age, sex, occlusion site, onset-to-needle time, onset-to-groin time, anesthesia type, and number of device passes. Edaravone Dexborneol treatment was associated with a favorable shift in the mRS distribution (common OR 1.98; 95% CI, 1.22–3.22; *p* = 0.005). The proportional odds assumption was tested and not violated (*p* = 0.384) (Table 5). These results are consistent with the binary logistic regression for mRS 0–2 and provide additional clinical granularity.

**Table 4:** Clinical outcomes at discharge and follow up.

Characteristics	Edaravone Dexborneol group (*n* = 110)	Control group (*n* = 97)	*p*-value
Efficacy outcomes at discharge			
mRS score 0–2, *n* (%)	57 (51.82)	34 (35.05)	0.017^*^
mRS score	2 (1–4)	4 (2–5)	0.056
Safety outcomes at discharge, *n* (%)			
All-cause mortality	7 (6.36)	13 (13.40)	0.102
Symptomatic ICH	3 (2.73)	4 (4.12)	0.720
Any ICH	8 (7.27)	10 (10.31)	0.468
HI/PH	6 (5.45)	7 (7.22)	0.775
PH-2	3 (2.73)	4 (4.12)	0.720
SAH	2 (1.82)	3 (3.09)	0.674
Early neurological deterioration	6 (5.45)	9 (9.28)	0.280
Pneumonia	12 (10.91)	14 (14.43)	0.402
Deep vein thrombosis	2 (1.82)	3 (3.09)	0.672
Pulmonary embolism	1 (0.91)	1 (1.03)	1.000
Renal dysfunction	5 (4.55)	6 (6.19)	0.571
Hypersensitivity reactions	1 (0.91)	0 (0.00)	0.315
Efficacy outcomes at 90 days follow-up			
mRS score 0–2, *n* (%)	74 (67.27)	45 (46.39)	0.003^**^
mRS score	2 (1–4)	3 (1–5)	0.018
Safety outcomes at 90 days follow-up, *n* (%)			
All-cause mortality, *n* (%)	9 (8.18)	14 (14.43)	0.186
Symptomatic ICH	4 (3.64)	5 (5.15)	0.754
Any ICH	12 (10.91)	15 (15.46)	0.337
HI/ PH	7 (6.36)	9 (9.28)	0.412
PH-2	5 (4.55)	8 (8.25)	0.390
SAH	3 (2.73)	5 (5.15)	0.478
Cerebral hernia	4 (3.64)	7 (7.22)	0.354
Respiratory failure	6 (5.45)	9 (9.28)	0.421
Acute ischemic stroke	3 (2.73)	4 (4.12)	0.708
Cerebral edema	7 (6.36)	11 (11.34)	0.226
Sudden cardiac death	2 (1.82)	4 (4.12)	0.422
Gastrointestinal hemorrhage	4 (3.64)	6 (6.19)	0.520
Liver dysfunction	4 (3.64)	4 (4.12)	1.000

Mean values ± standard deviation, median (interquartile range), and *n* (%) were reported for variables, respectively. mRS, modified Rankin Scale; ICH, intracranial hemorrhage; HI, hemorrhagic infarction; PH, parenchymal hematoma; SAH, subarachnoid hemorrhage.

^*^Absolute risk difference (ARD): 16.77%; 95% confidence intervals (CI): 3.44–30.09. Odds ratio (OR): 1.99; 95% CI: 1.14–3.49.

^**^ARD: 20.88%; 95% CI: 7.64–34.13. OR: 2.38; 95% CI: 1.35–4.18.

**Table 5:** Multivariable regression analyses for 90-day functional outcomes.

Variable	Binary logistic regression (mRS 0–2)			Ordinal logistic regression (mRS 0–6)		
Characteristics	Adjusted OR	95% CI	*p*-value	Common OR	95% CI	*p*-value
Age	0.971	0.943–0.999	0.028	0.976	0.953–0.999	0.041
Sex	0.631	0.342–1.162	0.135	0.853	0.559–1.302	0.448
Edaravone Dexborneol	2.134	1.142–3.985	0.018	1.982	1.221–3.216	0.005
ICA occlusion	0.381	0.199–0.726	0.004	0.462	0.284–0.750	0.002
Onset-to-needle	0.994	0.978–1.011	0.279	0.995	0.981–1.010	0.508
Onset-to-puncture	0.988	0.974–0.999	0.011	0.989	0.979–0.999	0.028
General anesthesia	0.722	0.372–1.398	0.327	0.743	0.428–1.289	0.291
Number of device passes	0.639	0.456–0.896	0.210	0.713	0.541–0.940	0.216

CI, confidence interval; OR, odds ratio. mRS, modified Rankin Scale; ICA, internal carotid artery.

Binary logistic regression reports adjusted ORs for functional independence (mRS 0–2 at 90 days).

Ordinal logistic regression reports common ORs for the full distribution of mRS scores (0–6).

Proportional odds assumption was tested and not violated (*P* = 0.384).

### Safety outcomes

All-cause mortality was numerically lower both in-hospital and at 90 days, without an increase in major safety events, including symptomatic intracranial hemorrhage. Expanded safety analysis showed no significant differences between groups across major complications (Table 4). Rates of any ICH, symptomatic ICH, PH-2 hematoma, and subarachnoid hemorrhage did not differ significantly. Similarly, the frequency of END, renal dysfunction, and hypersensitivity reactions was low and evenly distributed between groups. In-hospital complications such as pneumonia, DVT, and PE occurred infrequently and with no between-group differences. No unexpected adverse safety signals were observed with Edaravone Dexborneol.

## Discussion

This study provides novel clinical evidence that supports the neuroprotective and anti-inflammatory potential of Edaravone Dexborneol in AIS patients undergoing combined IVT and MT. This combination drug provides dual neuroprotection through free radical scavenging (Edaravone) and anti-inflammatory actions (Dexborneol). Our primary findings demonstrated that patients treated with Edaravone Dexborneol exhibited significantly better functional outcomes, with a higher proportion achieving a mRS score of 0–2 both at hospital discharge and at 90 days follow-up, compared to those receiving standard care alone. These improvements were accompanied by significantly lower levels of pro-inflammatory cytokines (IL-1β, IL-6, TNF-α) and elevated levels of anti-inflammatory cytokines (IL-10, IL-35, TGF-β) at multiple time points, suggesting that Edaravone Dexborneol’s neuroprotective benefit may be mediated through modulation of the inflammatory response.

Recent clinical evidence has increasingly highlighted the efficacy of Edaravone Dexborneol in acute ischemic stroke. A recent meta-analysis pooling data from randomized and observational studies demonstrated that the combination formulation is associated with improved functional outcomes and reduced inflammatory markers, with a favorable safety profile (17). Our findings are consistent with these results and add evidence in the context of patients undergoing MT combined with IVT.

Previous clinical studies have emphasized the importance of neuroprotection in AIS management. Despite successful large-vessel recanalization, many patients experience limited functional recovery due to microvascular injury, oxidative stress, and secondary neuronal damage (18, 19). Reperfusion therapies such as MT and IVT restore vessel patency but do not directly address downstream neuroinflammation, which remains a critical determinant of patient outcomes (20, 21). Hence, pharmacological adjuncts that can attenuate inflammation and reduce reperfusion injury are increasingly sought after.

Inflammation plays a pivotal role in ischemic brain injury. The release of pro-inflammatory cytokines, activation of microglia, and disruption of the blood–brain barrier (BBB) contribute to neuronal apoptosis and edema formation (22). Particularly, elevated IL-6 and TNF-α levels have been independently associated with worse functional outcomes in stroke patients, even in the presence of technically successful thrombectomy (23). Our findings support this pathophysiological link and further highlight that suppressing these cytokines—while enhancing anti-inflammatory mediators such as IL-10 and TGF-β—may facilitate better neurological recovery.

Importantly, prior trials of Edaravone monotherapy (e.g., standard 30 mg q12h regimens) should be distinguished from those evaluating Edaravone Dexborneol, as the latter provides dual mechanisms—free radical scavenging and anti-inflammatory effects via borneol (7, 24). While preclinical studies have demonstrated antioxidative and anti-inflammatory properties of both Edaravone and Edaravone Dexborneol, these results should be interpreted as mechanistic insights rather than direct clinical evidence (25). Our conclusions are drawn from human clinical data, and future randomized controlled trials will be required to confirm the efficacy of Edaravone Dexborneol in the MT + IVT setting.

Edaravone Dexborneol has long been recognized for its potent free radical scavenging ability. Preclinical studies in rodent models of cerebral ischemia–reperfusion injury have demonstrated that Edaravone Dexborneol reduces infarct volume, alleviates cerebral edema, and downregulates proinflammatory mediators such as IL-1β and MMP9 (26). Moreover, Edaravone Dexborneol modulates the immune response by inhibiting microglial activation and promoting the release of protective cytokines like IL-10 (27). While these mechanisms have been well validated in animal models, clinical translation has been limited. Our current study bridges this gap by providing real-world evidence in a clinical setting, showing that Edaravone Dexborneol effectively modulates inflammatory pathways in AIS patients undergoing reperfusion therapy.

This study suggests that Edaravone Dexborneol is a promising adjunct therapy for patients with AIS undergoing MT and IVT. By mitigating inflammatory responses during the acute phase, Edaravone Dexborneol may enhance neuroprotection and improve both short- and medium-term functional recovery. Further large-scale prospective trials are warranted to validate these findings and explore optimal timing and dosing strategies. In accordance with current stroke guidelines (AHA/ASA 2019, ESO 2021, Chinese Stroke Society 2023) (25, 28, 29), we ensured consistency in describing therapeutic windows for IVT (≤4.5 h) and MT (≤6 h, with possible extension by DAWN/DEFUSE-3 criteria), which are aligned with our study’s inclusion criteria. Nevertheless, given the retrospective observational design, causality cannot be inferred, and randomized controlled trials are warranted to validate these associations in the MT + IVT context.

### Limitations

This study has several limitations. First, it is a single-center, retrospective analysis, which may introduce inherent selection bias and limits the generalizability of the findings to broader populations. Second, although we applied multivariate adjustment to control for confounding variables, the retrospective design inherently restricts our ability to account for all potential confounders. Third, the sample size, while sufficient to identify statistical significance in many endpoints, may not be large enough to detect more subtle treatment effects or rare adverse events. Fourth, inflammatory biomarkers were measured at predefined time points, and dynamic fluctuations between these intervals may have been missed. Fifth, while our findings suggest a potential anti-inflammatory mechanism of Edaravone Dexborneol, causality cannot be firmly established without prospective randomized controlled trials. Sixth, treatment allocation to Edaravone Dexborneol appears non-random without explicit clinical criteria for use, which may introduce additional selection bias. Seventh, treatment allocation in this study was determined by the attending physicians rather than through randomization. This may have introduced potential selection bias, as physicians’ clinical judgment and patient characteristics could have influenced the choice of dexmedetomidine administration. Finally, this study did not investigate long-term functional and cognitive outcomes beyond 3 months, which may provide further insights into the sustained neuroprotective effects of Edaravone Dexborneol.

## Conclusion

Patients treated with Edaravone Dexborneol were associated with better functional outcomes three months after stroke onset compared to those who did not receive the treatment. These findings may be related to the drug’s anti-inflammatory properties; however, randomized controlled trials are needed to confirm efficacy in patients undergoing MT combined with IVT.

## Data Availability

The raw data supporting the conclusions of this article will be made available by the authors, without undue reservation.
